# *hsa-miR-320a-3p* and *hsa-miR-483-5p* levels in human granulosa cells: promising bio-markers of live birth after IVF/ICSI

**DOI:** 10.1186/s12958-022-01037-7

**Published:** 2022-11-21

**Authors:** Yu Liu, Qiaojuan Mei, Jiahao Yang, Qiuzi Shen, Min Zou, Jiao Li, Huaibiao Li, Ling Zhang, Wenpei Xiang

**Affiliations:** 1 Department of Gynecology, Maternal and Child Health Hospital of Hubei Province, Wuhan, 430070 P. R. China; 2 Institute of Reproductive Health and Center for Reproductive Medicine, Tongji Medical College, Huazhong University of Science and Technology, Wuhan, 430030 P. R. China

**Keywords:** *hsa-miR-320a-3p*, *hsa-miR-483-5p*, Human granulosa cells, Good-quality embryo, Live birth

## Abstract

**Background:**

MicroRNAs (miRNAs) are considered potential biomarkers for various diseases. This study investigated whether *hsa-miR-320a-3p* and *hsa-miR-483-5p* levels in human ovarian granulosa cells derived from follicular fluids are associated with embryo developmental competence.

**Methods:**

We collected 195 granulosa cells samples and analyzed the treatment outcomes in patients undergoing in vitro fertilization (*n* = 147) or intracytoplasmic sperm injection (*n* = 48) cycles. The *hsa-miR-320a-3p* and *hsa-miR-483-5p* levels in granulosa cells were measured using quantitative reverse transcription-polymerase chain reaction.

**Results:**

Patients were subdivided into four groups according to the granulosa cells *hsa-miR-320a-3p* and *hsa-miR-483-5p* levels quartiles (Q1–Q4). Embryo developmental competence was compared using the chi-square test. Patients in Q3 were less likely to achieve a normal fertilization rate for in vitro fertilization and blastocyst formation than those in Q1 as they expressed high levels of *hsa-miR-320a-3p* and *hsa-miR-483-5p* (*P* < 0.05). Patients in Q3 and Q4 were less likely to achieve a good-quality embryo as they expressed high levels of *hsa-miR-483-5p* and *hsa-miR-320a-3p* (*P* < 0.05). The *hsa-miR-320a-3p* and *hsa-miR-483-5p* levels were not associated with clinical pregnancy. However, multiple regression analysis indicated that in Q3 and Q4 intervals had experienced a decreased chance of live birth due to high expression levels of *hsa-miR-320a-3p* and *hsa-miR-483-5p* levels. The relative *hsa-miR-320a-3p* expression levels in granulosa cells were weakly and positively correlated with the patient age (*P* = 0.0033). Moreover, both the basal follicle stimulating hormone (*P* = 0.0003) and ovarian stimulation protocols (*P* = 0.006 and *P* = 0.004) significantly and positively affected *hsa-miR-320a-3p* levels. The days of stimulation was negatively correlated with the relative *hsa-miR-320a-3p* expression level (*P* = 0.047).

**Conclusions:**

The *hsa-miR-320a-3p* and *hsa-miR-483-5p* levels in human granulosa cells negatively correlated with the good-quality embryo rate and live birth, indicating that *hsa-miR-320a-3p* and *hsa-miR-483-5p* can be used as potential negative indicators to predict good-quality embryos and live births.

## Key message

*hsa-miR-320a-3p* in human granulosa cells can be used as a potential indicator to predict good-quality embryos and live births.

## Background

MicroRNAs (miRNAs) are highly conserved, single-stranded, small, non-coding, and functional RNAs of 19–25 nucleotides, which regulate post-transcriptional RNA levels by binding to the 3′-untranslated region of messenger RNAs (mRNAs) and causing destabilization or translation repression [1]. They are widely expressed in various biological systems. Although many miRNAs are commonly expressed, the selective specific expression of miRNAs is common in tissues, suggesting that the requirement for specific miRNAs in different tissues and specific roles of miRNAs in tissues. Owing to their tissue-specific expression, miRNAs are considered potential biomarkers [2].

Several studies have identified miRNAs that are expressed in ovarian follicle cells [3]. MiRNAs are involved in the regulation of various biological processes, including granulosa cell proliferation, apoptosis [4, 5], and oocyte maturation [5, 6]. Recent studies have reported that the miRNAs expression led to downstream events that will affected fertilization and day 3 embryo morphology [7]. Moreover, miRNAs could be promising biomarkers for ovarian responses during in vitro fertilization (IVF) [8]. Some miRNAs are also differentially expressed according to the fertilization method, chromosomal status, and pregnancy outcome, making them potential biomarkers for predicting IVF success [9]. These findings suggest that miRNAs play important roles in the oocyte development.

The aim of this study was to investigate the relationship between miRNAs (*hsa-miR-320a-3p* and *hsa-miR-483-5p*) in human granulosa cells expression levels and oocyte developmental competence and explored the effect of patient clinical characteristics on miRNAs (*hsa-miR-320a-3p* and *hsa-miR-483-5p*) expression levels in human granulosa cells.

## Materials and methods

### Patients’ characteristics

This study recruited 195 women enrolled in IVF (*n* = 147) or ICSI (*n* = 48) cycles at the Center for Reproductive Medicine of Tongji Medical College in the Huazhong University of Science and Technology from December 2019 to January 2021. Participants were required to meet the following eligibility requirements: conventional controlled stimulation protocols were used. Patients were excluded if they were diagnosed with infectious disease, malignant tumors, premature ovarian failure, polycystic ovary syndrome, systemic diseases and hereditary diseases. The women’s ages ranged from 21 to 46 years (mean ± SD: 34.39 ± 5.19 years) and their body mass index (BMI) ranged from 15.80 to 32.40 kg/m^2^ (mean ± SD: 22.60 ± 3.23 kg/m^2^). Baseline hormonal levels including follicle-stimulating hormone (FSH), luteinizing hormone (LH), and 17β-estradiol (E2) and anti-Mülerian hormone (AMH) were measured on the third day of menstruation. The number of days of stimulation ranged from 5 to 22 days (mean ± SD: 9.97 ± 2.48 days), and the total dose of gonadotropins received ranged from 900 to 6450 IU (mean ± SD: 2344.27 ± 842.52 IU).

The controlled ovarian stimulation protocols were used included ultra-long protocol, long protocol, antagonist protocol, progestin-primed ovarian stimulation (PPOS), mild stimulation protocol, and luteal phase stimulation. FSH stimulation was monitored by measuring serum E2 levels and follicular size. Human chorionic gonadotrophin (hCG) (Livzon, Zhuhai, China) was injected when at least three follicles are 18 mm or larger in diameter by ultrasound. After hCG injection 36 h, oocytes were extracted by transvaginal ultrasound-guided puncture.

### Human granulosa cells collection and identification

Granulosa cells were collected from the follicular fluid of 195 patients as described [10]. Briefly, after the isolation of the cumulus-oocyte complexes (COCs) for conventional IVF or ICSI procedures, the follicular fluids were centrifuged and granulosa cells were collected and resuspended in 1× phosphate-buffered saline (PBS). Then, it was added to a 50% Percoll gradient (GE Healthcare Life Sciences, Piscataway, NJ, USA) and centrifuged at 400 g for 30 min at 4 °C. The cells in the middle layer were collected, resuspended in PBS.

To confirm the purity of granulosa cells, it was seeded and cultured on coverslips at a density of 1 × 10^5^ cells/ coverslips for 48 h. Then the granulosa cells were fixed in 4% (v/v) paraformaldehyde for 20 min for immunofluorescence as before [11]. The FSH receptor (FSHR) was used to detect the purity of granulosa cells. To exclude the non-specific staining from antibodies, the primary and secondary antibodies were omitted as negative control groups, respectively.

### RNA isolation, cDNA synthesis, and real-time quantitative PCR (qPCR)

Total RNA was extracted from granulosa cells using the RNA-easy Isolation Reagent (Vazyme Biotech Co., Ltd., Nanjing), and transcribed into cDNA using the All-in-One™ miRNA quantitative reverse transcription-polymerase chain reaction (qRT-PCR) Detection Kit 2.0 (GeneCopoeia, Inc., United States) according to the manufacturer’s protocol. The cDNA synthesis reaction conditions were the following: 37 °C for 60 min and 85 °C for 5 s.

The *hsa-miR-320a-3p* and *hsa-miR-483-5p* primers were purchased by the GeneCopoeia Company. *hsa-miR-320a-3p* primer forward:5′-TTGAGAGGGCGAAAAAAA-3′. *hsa-miR-483-5p* primer forward: 5′-CGGGAGGAAAGAAGGGAGAA-3′. Reverse primers are universal reverse primers in the All-in-One™ miRNA qRT-PCR Detection Kit (GeneCopoeia, Inc. USA). U6 was used as a housekeeping gene. The reaction was performed in a total volume of 20 μL contained 10 μL 2× All-in-One™ qPCR Mix, 2 μL All-in-One™ miRNA qPCR Primer (2 μM), 2 μL Universal Adaptor PCR Primer (2 μM) and 2 μL First-strand cDNA. The cycling conditions used were the following: 95 °C for 600 s, 40 cycles at 95 °C for 10 s, 60 °C for 20 s and 72 °C for 10 s. The relative quantity of miRNA expression was calculated using the 2^−△△CT^ method.

### Morphological assessment of oocytes, good-quality embryos, and blastocysts

The appearance of prokaryotic zygote 18 to 20 hours after microinjection or artificial insemination is a representative of fertilization. IVF normal fertilization rate = number of 2PN/total number of oocytes × 100%. ICSI normal fertilization rate = number of 2PN/total number of MII oocytes × 100%. Morphological scores of embryos at day 3 were consistent with the current consensus system [12]. Good-quality embryos and blastocysts were defined as previous [13]. Good-quality embryo rate = number of day 3 good-quality embryos/normal fertilization number of cleavage embryos × 100%. Blastulation rate = number of blastocysts at stage 2 and above/total number of cleavage embryos in blastocyst culture × 100%.

### Statistical analysis

The *hsa-miR-320a-3p* and *hsa-miR-483-5p* levels, expressed as means ± standard deviation (SD), median values and the interquartile range (IQR), or as OR (95% CI), if appropriate. Linear regression was carried out for the effect of patients’ characteristics information on the *hsa-miR-320a-3p* and *hsa-miR-483-5p* levels in human granulosa cells. To evaluate the correlation between *hsa-miR-320a-3p* and *hsa-miR-483-5p* levels and embryo developmental competence, we first subdivided all 195 samples according to their granulosa cells *hsa-miR-320a-3p* and *hsa-miR-483-5p* levels quartile, then the normal fertilization rate, good-quality embryo rate and blastulation rate were compared by Chi-square test. Multi-variable logistic regression analysis was used to analyze clinical pregnancy and live birth. Statistical analyses were performed using the Statistical Package for Social Sciences program, Version 12.0 (SPSS Inc., Chicago, IL, USA). *P* < 0.05 was considered statistically significant.

## Results

### Identification of granulosa cells from human follicular fluids

**Fig. 1:**
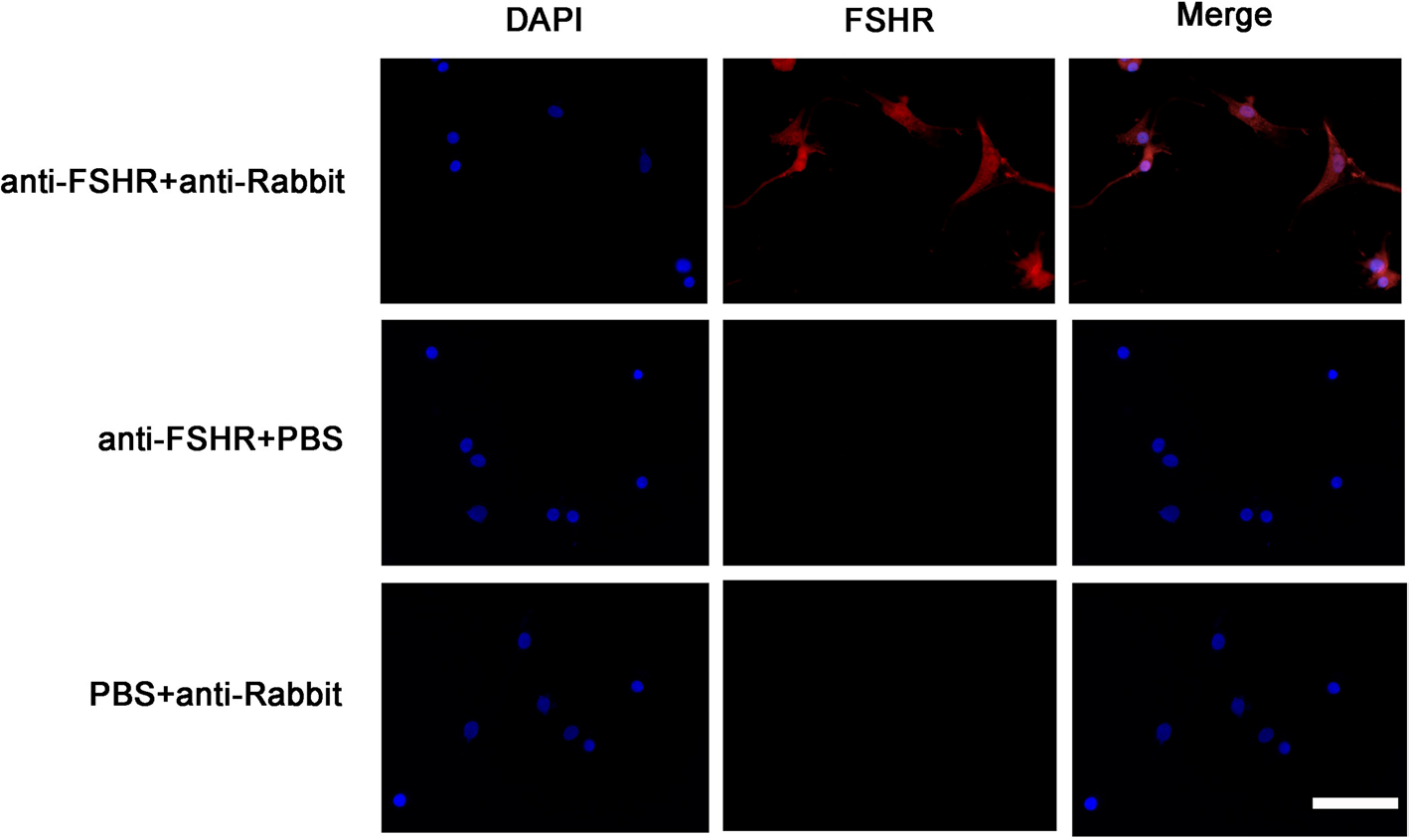
A representative image of immunofluorescence staining in human ovarian granulosa cells (*n* = 5). The Red (× 400) expressed FSHR, the blue (× 400) expressed nuclear staining using 4 ‘, 6-diamino-2-phenylindole (DAPI). Non-specific staining can be observed with PBS instead of primary or secondary antibodies

As shown in Fig. 1, the 96% cells in the dishes were granulosa cells, which were characterized by a positive FSHR staining. Non-specific staining was not detected. This proves that all the cells isolated were granulosa cells, and directly extracted granulosa cells can be used in subsequent studies.

### Relationship of the *hsa-miR-320a-3p* and *hsa-miR-483-5p* levels in the human granulosa cells and embryo developmental competence

The patients were subdivided into four groups according to the relative expression of *hsa-miR-320a-3p* levels quartile in the granulosa cells: Q1: 0.46-6.17 × 10^3^, *n* = 49; Q2: 6.41 × 10^3^-2.35 × 10^5^, *n* = 49; Q3: 2.63 × 10^5^-2.34 × 10^6^, *n* = 49; and Q4: 2.51 × 10^6^-9.38 × 10^7^, *n* = 48. In the Q3 intervals, the normal fertilization rate for IVF was lower compared to Q1 and Q2 (Table 1, *P* < 0.05). In the Q3 and Q4 intervals, the good-quality embryo rate was lower than Q2 (Table 1, *P* < 0.0001). However, the normal fertilization rate for ICSI and blastulation rate did not differ (Table 1, *P* > 0.05). Multiple regression analysis showed that in Q3 and Q4 intervals experienced a decreased chance of live birth when Q1 group was used as reference (Table 2, *P* < 0.0001). And there was no difference in clinical pregnancy (Table 2, *P* > 0.05).

The patients were subdivided into four groups according to the relative expression of *hsa-miR-483-5p* levels quartile: Q1: 0.002-0.18, *n* = 49; Q2: 0.18-1.13, *n* = 49; Q3:1.21-5.80, *n* = 49; and Q4: 5.81-3.52 × 10^3^, *n* = 48, respectively. In the Q3 intervals, the good-quality embryo rate and blastulation rate were lower than Q1 group (Table 1, *P* < 0.05). The normal fertilization rate for IVF or ICSI was no significant differences among groups (*P* > 0.05), as shown in Table 1. Multiple regression analysis showed that in Q3 and Q4 intervals had a decreased chance of live birth (Table 2, *P* < 0.0001). Clinical pregnancy had no differ among four group (Table 2, *P* > 0.05).

**Table 1:** Association between the levels of *hsa-miR-320-3p* and *hsa-miR-483-5p* in granulosa cells from human follicular fluids and reproductive outcomes of assisted reproductive technology (ART)

Parameters									*P*-value
	Q1	%	Q2	%	Q3	%	Q4	%	
*hsa-miR-320a-3p*									
Normal fertilization rate									
IVF cycles	201/358	56.1	201/361	55.7	203/405	50.1	225/368	61.1	< 0.05
ICSI cycles	95/131	72.5	74/96	77.1	93/142	65.5	37/61	60.7	NS
Good-quality embryo rate	212/296	71.6	205/275	74.5	214/296	72.3	149/262	56.9	< 0.0001
Blastulation rate	81/212	38.2	65/205	31.7	82/214	38.3	55/149	36.9	NS
*hsa-miR-483-5p*									
Normal fertilization rate									
IVF cycles	167/341	49.0	258/457	56.5	173/346	50.0	232/348	66.7	NS
ICSI cycles	105/134	78.4	52/74	70.3	83/133	62.4	59/89	66.3	NS
Good-quality embryo rate	225/272	82.7	203/310	65.5	197/256	77.0	155/291	53.3	< 0.0001
Blastulation rate	86/225	38.2	78/203	38.4	55/197	27.9	64/155	41.3	< 0.05

Chi square test was used for statistical analysis

*Q* quartile, *NS* not statistically significant, *2PN* two pronuclei

**Table 2:** Multi-variable logistic regression analysis of the levels of *hsa-miR-320a-3p* and *hsa-miR-483-5p* in human granulosa cells and clinical outcomes (OR 95% CI)

	Clinical pregnancy		Live birth	
	*hsa-miR-320a-3p*	*hsa-miR-483-5p*	*hsa-miR-320a-3p*	*hsa-miR-483-5p*
Q1	Reference	Reference	Reference	Reference
Q2	0.328 (0.011-10.20)	7.08 (0.17-297.02)	5.24 (8.58 × 10^−5^-3.20 × 10^5^)	0.21 (1.97 × 10^−8^-2.12 × 10^6^)
Q3	0.069 (0.001-5.46)	0.89 (0.01-84.12)	0.93 (2.14 × 10^−5^-4.04 × 10^4^)	0.096 (2.57 × 10^−11^-3.61 × 10^8^)
Q4	1.59 (0.08-33.53)	11.23 (0.54-234.83)	0.375 (1.17 × 10^− 12^-1.21 × 10^11^)****	0.163 (7.57 × 10^− 14^-3.53 × 10^11^)****

****P < 0.0001

### Effect of patients’ clinical characteristics on the *hsa-miR-320a-3p* and *hsa-miR-483-5p* levels in the human granulosa cells

The relative expression of *hsa-miR-320a-3p* in the human granulosa cells were weak positively correlated with age (*β* ± SE: 4.79 × 10^5^ ± 1.61 × 10^5^, *P* = 0.0033) (Table 3). Moreover, both the basal FSH (*β* ± SE: 7.90 × 10^5^ ± 2.14 × 10^5^, *P* = 0.0003) (Table 3) and ovarian stimulation protocol, including mild stimulation protocol and luteal phase stimulation (*β* ± SE: 8.27 × 10^− 9^ ± 2.92 × 10^− 9^, 6.29 × 10^− 9^ ± 2.09 × 10^− 9^, respectively; *P* = 0.006, *P* = 0.004, respectively) (Table 3) significantly and positively affected *hsa-miR-320a-3p* levels in the human granulosa cells. The days of stimulation were negatively correlated with the relative expression of *hsa-miR-320a-3p* in the human granulosa cells (*β* ± SE: − 6.85 × 10^5^ ± 3.42 × 10^5^, *P* = 0.0466) (Table 3). The relative expression of *hsa-miR-320a-3p* in the human granulosa cells were not associated with BMI, basal LH, basal E_2_, AMH, AFC and total dose of gonadotropins (Table 3, *P* > 0.05).

The relative expression of *hsa-miR-483-5p* in the human granulosa cells were not associated with age, BMI, female baseline levels, AFC, days of stimulation, total dose of gonadotropins and ovarian stimulation protocol (Table 3, *P* > 0.05).

**Table 3:** Patients’ characteristics association with the *hsa-miR-320a-3p* and the *hsa-miR-483-5p* levels in granulosa cells from human follicular fluids

Variable	Min-Max	Mean	n (%)	SD	*hsa-miR-320a-3p*		*hsa-miR-483-5p*	
					*β* ± SE	*P-*value	*β* ± SE	*P-*value
Age (years)	21-46	34.39	195 (100)	5.19	4.79 × 10^5^ ± 1.61 × 10^5^	0.0033*	−3.58 ± 3.51	0.3084
BMI (kg/m^2^)	8.93-32.40	22.53	194 (99.49)	3.39	1.45 × 10^5^ ± 2.54 × 10^5^	0.5678	−1.48 ± 5.43	0.7850
Female baseline levels								
Basal FSH (IU/L)	1.25-33.00	8.40	195 (100)	3.86	7.90 × 10^5^ ± 2.14 × 10^5^	0.0003*	−3.42 ± 4.72	0.4701
Basal LH (IU/L)	0.65-40.02	4.76	195 (100)	3.77	7.61 × 10^3^ ± 2.27 × 10^5^	0.9733	−0.97 ± 4.85	0.8412
Basal E_2_ (pg/ml)	2.74-5178	76.17	195 (100)	370.11	−6.09 × 10^2^ ± 2.3 × 10^3^	0.7926	−7.57 × 10^−4^ ± 4.94 × 10^−2^	0.9878
AMH (ng/ml)	0.06-14.62	3.52	194 (99.49)	2.93	−1.91 × 10^5^ ± 2.93 × 10^5^	0.5147	−3.917 × 10^− 3^ ± 6.039 × 10^− 3^	0.5174
Antral follicle count	3-52	15.74	195 (100)	7.97	−5.39 × 10^4^ ± 1.07 × 10^5^	0.6159	−0.13 ± 2.30	0.9535
Days of stimulation	5-22	9.97	195 (100)	2.48	−6.85 × 10^5^ ± 3.42 × 10^5^	0.0466*	0.31 ± 7.38	0.9660
Total dose of gonadotropins (IU)	900-6450	2344.27	195 (100)	842.52	−1.39 × 10^3^ ± 1.01 × 10^3^	0.1704	−7.40 × 10^−3^ ± 2.17 × 10^−2^	0.7333
Ovarian stimulation protocol								
Ultra-long protocol	–	–	62 (31.79)	–				Ref
Long protocol	–	–	9 (0.05)	–	−6.05 × 10^−9^ ± 5.36 × 10^−9^	0.263	−2.57 × 10^−5^ ± 9.61 × 10^− 5^	0.790
Antagonist protocol	–	–	75 (0.38)	–	−7.10 × 10^−9^ ± 6.15 × 10^− 9^	0.250	−1.76 × 10^−4^ ± 1.42 × 10^− 4^	0.217
Progestin-primed ovarian stimulation (PPOS)	–	–	40 (0.21)	–	2.19 × 10^− 9^ ± 4.37 × 10^− 9^	0.617	−1.30 × 10^− 4^ ± 1.40 × 10^− 4^	0.354
Mild stimulation protocol	–	–	5 (0.03)	–	8.27 × 10^− 9^ ± 2.92 × 10^− 9^	0.006*	−1.44 × 10^− 5^ ± 7.61 × 10^− 5^	0.851
Luteal phase stimulation	–	–	4 (0.02)	–	6.29 × 10^− 9^ ± 2.09 × 10^− 9^	0.004*	−1.93 × 10^− 5^ ± 6.91 × 10^− 5^	0.781

*P < 0.05

## Discussion

In this study, our results indicated that *hsa-miR-320a-3p* and *hsa-miR-483-5p* expression levels in the human granulosa cells were negatively associated with good-quality embryos and live births. Moreover, *hsa-miR-483-5p* levels were negatively associated with blastulation. Further studies revealed that *hsa-miR-320a-3p* levels positively correlated with patient age and basal follicle stimulating hormone (FSH) levels.

Follicular fluid content can be used as a noninvasive marker to predict oocyte quality. In our study, we found that a significantly difference in normal fertilization rate those with high *hsa-miR-320a-3p* expression levels during IVF cycles. Further, multi-variable logistic regression analysis indicated that the high expression levels of *hsa-miR-320a-3p* and *hsa-miR-483-5p* in granulosa cells seemed to reduce the number of good-quality embryos and live births (*P* < 0.0001). Notably, patients with higher levels of *hsa-miR-483-5p* exhibited a decreasing trend in blastulation. These results suggested a negative effect of *hsa-miR-320a-3p* and *hsa-miR-483-5p* on oocyte development and pregnancy outcomes. Additionally, *hsa-miR-320a-3p* and *hsa-miR-483-5p* have been reported to plays important roles in inhibiting cell proliferation and migration [14–16]. These processes have been proven to affect oocyte development [17]. *hsa-miR-320a-3p* and *hsa-miR-483-5p* in granulosa cells may also acts as apoptosis factors and decrease oocyte development via a paracrine mechanism. Consequently, higher levels of *hsa-miR-320a-3p* and *hsa-miR-483-5p* in granulosa cells may reduce the developmental competency of oocytes.

Furthermore, our results suggested that *hsa-miR-320a-3p* expression is weakly and positively correlated with patient age (r^2^ = 0.209, *P* = 0.0033). Ansere et al. revealed that cellular senescence may contribute to ovarian aging, and the subsequent decline in ovarian follicular reserve [18]. In our study, the *hsa-miR-320a-3p* levels were positively associated with basal FSH levels (r^2^ = 0.257, *P* = 0.0003). It is a useful predictor of ovarian reserve [19, 20], indicating an association between *hsa-miR-320a-3p* and ovarian reserve function. However, no obviously relationship was observed between *hsa-miR-320a-3p* and AMH, AFC or BMI, which have also been reported to affect ovarian functions [21, 22]. These conflicting results may be due to several factors, such as the cause of infertility and ovarian stimulation protocols. The potential associations between *hsa-miR-320a-3p* and FSH may provide a new direction to predict ovarian reserve function.

Many positive regulatory indicators predict the ART outcomes in human granulosa cells, such as circRNA [23], AQP7 [24] and telomerase activity [25]. As negative regulatory indicators, *hsa-miR-320a-3p* and *hsa-miR-483-5p,* can be combined with positive regulatory indicators to make the prediction results more reliable.

## Conclusion

The current study indicated that the expression levels of *hsa-miR-320a-3p* and *hsa-miR-483-5p* in granulosa cells are negatively associated with good-quality embryos and live births in women undergoing IVF**/**ICSI. Notably, patients with higher levels of *hsa-miR-483-5p* exhibited a decreasing trend in blastulation. These results suggest that *hsa-miR-320a-3p* and *hsa-miR-483-5p* could be used as potential indicators to predict the quality of embryos and live births.

## Data Availability

The datasets used and/or analysed during the current study are available from the corresponding author on reasonable request.

## Abbreviations

IVF: In vitro fertilization
ICSI: Intracytoplasmic sperm injection
BMI: Body mass index
FSH: Follicle-stimulating hormone
LH: Luteinizing hormone
E2: 17β-estradiol
AMH: Anti-Mülerian hormone
hCG: Human chorionic gonadotrophin
qRT-PCR: Quantitative reverse transcription-polymerase chain reaction
